# Baicalein improves the chemoresistance of ovarian cancer through regulation of CirSLC7A6

**DOI:** 10.1186/s13048-023-01285-0

**Published:** 2023-11-08

**Authors:** Shuqing Li, Zhihui Yi, Mingqing Li, Zhiling Zhu

**Affiliations:** Department of Obstetrics and Gynecology, Obstetrics and Gynecology Hospital of Fudan University, 128 Shenyang Road, Shanghai, 200090 China

**Keywords:** Ovarian cancer, Chemoresistance, Baicalein, CirSLC7A6, SLC7A6

## Abstract

**Purpose:**

The present study aimed to investigate whether baicalein improves the sensitivity of resistant ovarian cancer cells to cisplatin.

**Methods:**

Transcriptomic sequencing and bioinformatics analysis were used to screen differentially expressed CirSLC7A6 in A2780 and A2780/CDDP cells. RT-qPCR was performed to examine the expression levels of CirSLC7A6, miR-2682-5p, and SLC7A6. Cell proliferation and apoptosis were examined using a Cell Counting Kit-8 assay and flow cytometry, and cell migration and invasion were analyzed using wound healing and Transwell assays. Cell suspensions were inoculated into the subcutaneous tissues of the bilateral interscapular region of nude mice. Saline, cisplatin, baicalein and cisplatin plus baicalein were intraperitoneally injected to observe the effects on tumor growth. Toxicity analyses in the liver and kidney were performed using H&E staining. RT-qPCR and immunohistochemistry were used to detect the expression of CirSLC7A6, miR-2682-5p, and SLC7A6 in tumor tissues, and western blot analysis was carried out to measure protein expression levels.

**Results:**

CirSLC7A6 was markedly upregulated in A2780/CDDP cells compared with the A2780 cells. CirSLC7A6 knockdown notably increased the expression of miR-2682-5p and decreased SLC7A6 expression. The rates of inhibition and apoptosis in the group treated with a combination of cisplatin and baicalein were significantly higher than those of the cisplatin and baicalein groups of A2780/CDDP shCirSLC7A6 cells. In A2780/CDDP shCirSLC7A6 cells, migration and invasion were significantly higher in the cisplatin and baicalein groups, compared with the combined treatment group. In the A2780/CDDP shCirSLC7A6 cell xenograft, the tumor weight of the combined treatment group was significantly lower than that of the cisplatin and baicalein groups. In addition, the combination of cisplatin and baicalein did not induce higher levels of toxicity in the liver or kidney. Baicalein alone and in combination with cisplatin notably reduced the expression of CirSLC7A6 and SLC7A6, and increased the expression of miR-2682-5p in the A2780/CDDP shCirSLC7A6 cell xenograft. In A2780/CDDP shCirSLC7A6 cells, the expression levels of P-Akt, P-mTOR, P-Erk, Bcl-2 and MMP2 were lower in the combined treatment group than in the control group.

**Conclusions:**

Treatment with baicalein improved the sensitivity of ovarian cancer cells to cisplatin and inhibited cell proliferation, metastasis and tumor growth.

## Introduction

Recurrence and drug resistance are key issues in the clinical treatment of epithelial ovarian cancer (EOC) [1]. Therefore, further investigations into the mechanisms underlying chemoresistance and the development of targeted drugs will significantly improve the treatment options for ovarian cancer [2].

Baicalein exerts anti-inflammatory, antioxidant and antitumor pharmacological effects, and cooperates with chemotherapeutic drugs to serve anti-tumor effects [3–5]. Previous studies have demonstrated that baicalein combined with docetaxel induces apoptosis and inhibits metastasis of thyroid cancer cells through regulation of Akt/mTOR and Erk pathways [6]. Moreover, baicalein combined with cisplatin induces apoptosis of lung cancer cells via the regulation of Bcl-2, Bax and Caspase-3 [7]. In pancreatic cancer, the combination of baicalein and gemcitabine inhibits proliferation and induces apoptosis through inhibiting the expression of Bcl-2 and Survivin, and promoting the expression of Bax, Caspase-3 and PARP [8]. Combined treatment with baicalein and paclitaxel may induce apoptosis of ovarian cancer cells by increasing the release of cytochrome c, activating Caspase-3/-9 and inhibiting the Akt/β-catenin pathway [9].

Circular RNA (circRNA) possesses a post-transcriptional regulatory function and is rich in micro (mi) RNA binding sites [10]. MiRNA is involved in the formation of chemoresistance of ovarian cancer via the regulation of proliferative and apoptotic pathways, drug metabolism, and DNA damage repair [1, 11]. Circ_0078607 inhibits the proliferation and metastasis of ovarian cancer cells and promotes apoptosis via the regulation of the miR-32-5p/SIK1 axis [12]. In addition, CircFOXP1 improves cisplatin resistance in ovarian cancer and CircEXOC6B enhances the sensitivity of ovarian cancer cells to paclitaxel [13, 14].

Solute carrier family 7 (SLC7) are amino acid transporters [15]. Amino acid transporters are upregulated in tumor cells to facilitate the uptake of amino acids required for rapid proliferation [16]. Furthermore, amino acid transporters affect the transport of drugs, such as cisplatin and gemcitabine, in various tumor cells, resulting in increased drug excretion in cells and chemoresistance [17]. Therefore, blocking transporter activity may significantly inhibit cell proliferation and improve sensitivity to chemotherapeutic drugs [18–20]. Silencing SLC3A2 inhibits mTOR pathway activity and cell proliferation in cervical cancer cells [21]. In addition, SLC3A2 knockdown reduces the expression of Akt, ERK1/2 and matrix metalloproteinase (MMP) 2 and suppresses tumor progression and invasion in cervical cancer [22]. SLC7A5 is abnormally expressed in a variety of tumor cells and may act as a potential target for anticancer drugs [23]. CircZNF124 regulates the expression of SLC7A5 through binding to miR-199b-5p in endometrial cancer. Long non-coding (lnc) RNA OIP5-AS1 upregulates SLC7A5 via targeting miR-152-3p, resulting in increased leucine uptake, further promoting proliferation, migration and invasion of endometrial cancer cells. Silencing CircZNF124 and OIP5-AS1 prevents the progression of endometrial cancer [24, 25]. Notably, the effects of SLC7A6 on the chemoresistance of ovarian cancer are yet to be fully elucidated. Results of the present study uncovered a novel molecular mechanism that baicalein acts as a cisplatin sensitizer, via inhibition of CirSLC7A6/miR-2682-5p/SLC7A6 in ovarian cancer.

## Materials and methods

### Chemicals

Baicalein was purchased from Sigma-Aldrich (Merck KGaA, USA). Cisplatin was purchased from Hansoh (China).

### Cell lines and cell culture

The human epithelial ovarian cancer cell lines A2780 and A2780/CDDP were purchased from the American Type Culture Collection (ATCC). The normal human ovarian epithelial T29 cells were purchased from the ATCC. Cells were cultured in RPMI 1640 medium (Corning, Inc., USA) containing 10% fetal bovine serum (Gibco; Thermo Fisher Scientific, Inc., USA), 100 U/ml penicillin and 100 μg/ml streptomycin. All cells were incubated at 37 °C in a humidified atmosphere containing 5% CO_2_.

### Transcriptomic sequencing

Transcriptome sequencing was conducted by NovelBio Bio-Pharm Technology Co., Ltd., (China). Bioinformatics analysis was conducted using the Kyoto Encyclopedia of Genes and Genomes (KEGG) pathway database, The Human Gene Database and RNA22. Using these databases, circRNAs with significantly different expression levels were screened for in A2780 and A2780/CDDP cells.

### Cell transfection

A2780/CDDP cells were transfected with short hairpin (sh) CirSLC7A6 or shGFP, and A2780 cells were transfected with CirSLC7A6 or Vector (Shanghai GeneChem Co., Ltd., China) according to the manufacturer’s protocols. Briefly, A2780/CDDP and A2780 cells were transfected in six-well plates for 16 h. The medium was replaced, and 1 μg/ml puromycin (cat. no. ST551; Beyotime Institute of Biotechnology, China) was used to select stably transduced cells. Knockdown efficiency was confirmed using reverse transcription-quantitative polymerase chain reaction (RT-qPCR).

### RT-qPCR

Total RNA was extracted from cells/tissues using a Cell/Tissue miRNA Kit (cat. no. 19331ES50; Shanghai Yeasen Biotechnology Co., Ltd., China). Subsequently, RNA was reverse transcribed into cDNA using a Strand cDNA Synthesis Kit (cat. nos. 11121ES60 and 11141ES60; Shanghai Yeasen Biotechnology Co., Ltd., China). cDNA was amplified on a VeritiTM 96-well thermal cycler (Thermo Fisher Scientific, Inc., USA). RT-qPCR analysis was performed using a qPCR SYBR Master Mix (cat. no. 11184ES08; Shanghai Yeasen Biotechnology Co., Ltd., China). The expression of β-actin was used to normalize the expression of mRNA. qPCR was carried out using QuantStudioTM 6 Flex Real-Time PCR Systems (Thermo Fisher Scientific, Inc., USA). Relative mRNA expression was calculated using the 2-ΔΔCT method. Each sample condition contained three biological replicates, and data was presented as the mean ± standard deviation (SD). The experiment was performed for at least three technical replicates. The sequences of primers used in the present study are listed in Table 1.

**Table 1:** The sequences of primers

Gene	Primer sequences
ACTB	Forward: CCTGGCACCCAGCACAAT
	Reverse: GGGCCGGACTCGTCATAC
CirSLC7A6	Forward: GGGGCTTCATTGCCTTCATC
	Reverse: CCTGGGACTGGCTGGTGTTA
SLC7A6	Forward: AGGCCATTGTTTATTCGGAATG
	Reverse: TTTTTCTTCGGCTACATCAAGC

*circRNA *circular RNA, *SLC7 *Solute carrier family 7

### Cell viability

A Cell Counting Kit-8 (CCK-8) assay was carried out to evaluate the cytotoxicity of the treatments. Briefly, 4,000–5,000 cells/well were seeded into 96-well plates and cultured overnight. Cells were subsequently treated with cisplatin, baicalein or cisplatin plus baicalein for 48 h, followed by treatment with 10 μl CCK-8 reagent (cat. no. C0039; Beyotime Institute of Biotechnology, China). Following incubation for 2–4 h at 37 °C, samples were analyzed using a Synergy H1 multi-mode microplate reader (BioTek Instruments, Inc., USA). Each sample condition contained three biological replicates, and the experiment was performed for at least three technical replicates.

### Flow cytometry

Cell apoptosis was detected using APC Annexin V Apoptosis Detection Kit with 7-AAD (cat. no. 640930; BioLegend, Inc., USA) according to the manufacturer’s instructions. Briefly, cells were treated with cisplatin, baicalein or cisplatin plus baicalein for 48 h, harvested using trypsin digestion solution without EDTA and washed twice with PBS. Following detection of the blank control, the treated groups were resuspended in binding buffer and incubated with APC Annexin V and 7-AAD for 15 min at room temperature in the dark. Cells were analyzed using a CytoFLEX flow cytometer (Beckman Coulter, Inc., USA). Data analysis was performed using FlowJo software. The experiment was performed for at least three technical replicates.

### Wound healing assay

A total of 5 × 10^5^ cells/well were seeded into 6-well plates. When the cell density reached 80–90%, a scratch was made in the monolayer in the middle of the well using a 20 μl pipette tip. The tip was kept perpendicular to the bottom of the well to obtain a straight gap. Wound healing was subsequently observed and photographed at 48 h. The experiment was performed for at least three technical replicates.

### Transwell assay

Cell invasion was evaluated using Transwell chambers (Corning, Inc., USA) that were coated with BD Matrigel™ (cat. no. 354234; BD Biocoat, USA). The upper chamber contained cell cultures in serum-free medium, while the lower chamber was filled with complete medium. A total of 1 × 10^5^ cells were seeded into the upper chamber and treated with cisplatin, baicalein, or cisplatin plus baicalein for 48 h at 37 °C. Cells that invaded the lower surface of the chamber were fixed with 4% paraformaldehyde fix solution and stained with crystal violet staining solution. The number of invaded cells was counted using a microscope. The experiment was performed for at least three technical replicates.

### Tumor xenograft models

Experiments were reviewed and approved by the Institutional Animal Care and Use Committee of Obstetrics and Gynecology Hospital of Fudan University. Mice were maintained under specific-pathogen-free conditions, housed in isolated vented cages and handled using aseptic procedures. 4-week-old female BALB/c nude mice were randomly divided into 16 groups with six mice per group. Single cell suspensions from A2780, A2780/CDDP, A2780/CDDP shCirSLC7A6 or A2780/CDDP shGFP cells were injected (5.0 × 10^6^ cells/100 ul in phosphate buffered saline) subcutaneously into the bilateral interscapular region of the mice. Post-injection mice were randomized into four groups: Control, cisplatin, baicalein and cisplatin plus baicalein. When the tumor volume reached ~ 100 mm^3^, control mice were injected intraperitoneally with 0.9% sterile saline every other day. Cisplatin mice were injected intraperitoneally with 5 mg/kg cisplatin every three days. Baicalein mice were injected intraperitoneally with 20 mg/kg baicalein every other day. Cisplatin plus baicalein mice were injected intraperitoneally with 5 mg/kg cisplatin every three days and 20 mg/kg baicalein every other day. Tumor volume was measured every three days using a caliper. The following formula was used to calculate tumor volumes. Tumor volume (mm^3^) = (length x width^2^) × 0.52 (length x width^2^). Mice were sacrificed for tumor dissection using cervical dislocation. Liver and kidney tissues were dissected, paraffin-embedded, sectioned and stained using hematoxylin and eosin (H&E) staining. For immunohistochemistry, slides of tumors were incubated with anti-SLC7A6 (cat. no. ab235054; Abcam, UK).

### Western blotting

Cells were lysed in RIPA Lysis Buffer (cat. no. P0013B, Beyotime Institute of Biotechnology, China) with freshly added protease and phosphatase inhibitor cocktails (cat. no. 1050, Beyotime Institute of Biotechnology, China) and ultranuclease (cat. no. 20125ES25, Shanghai Yeasen Biotechnology Co., Ltd., China). Cells were subsequently centrifuged at 13,200 RPM at 4 °C for 15 min. Cell supernatants were harvested and quantified using a BCA Protein Assay Kit (cat. no. P0012; Beyotime Institute of Biotechnology, China) according to the manufacturer’s instructions. Electrophoresis was carried out using SDS-PAGE gel (cat. no. P0012AC, Beyotime Institute of Biotechnology, China) and transferred onto PVDF membranes (Millipore, USA). Membranes were blocked with 5% non-fat milk in Tris-buffered saline with Tween-20 (TBST) for 1 h at room temperature. Membranes were subsequently incubated with the following primary antibodies: Phosphorylated (P) -Erk1/2 (Thr202/Tyr204; cat. no. 4370; Cell Signaling Technology, Inc., USA), Erk1/2 (cat. no. 4376; Cell Signaling Technology, Inc., USA), P-p38 (Thr180/Tyr182; cat. no. 4511; Cell Signaling Technology, Inc., USA), p38 (cat. no. 8690; Cell Signaling Technology, Inc., USA), PI3 Kinase p85α (cat. no. 13666S; Cell Signaling Technology, inc., USA), P-Akt (Ser473; cat. no., 4060 T; Cell Signaling Technology, Inc., USA), Akt (cat. no. 4691 T; Cell Signaling Technology, Inc., USA), P-mTOR (Ser2448; cat. no., 5536 T; Cell Signaling Technology, Inc.), mTOR (cat. no., 2983 T; Cell Signaling Technology, Inc., USA), Bcl-2 (cat. no., 15071 T; Cell Signaling Technology, Inc., USA), SLC7A6 (cat. no. ab235054; Abcam, UK), recombinant anti-MMP2 (cat. no. ab97779; Abcam, UK), β-actin (cat. no., 3700 T; Cell Signaling Technology, Inc., USA) and Protein Marker (cat. nos. 26616 and 26,619; Thermo Fisher Scientific, Inc., USA). Membranes were incubated at 4 °C overnight and subsequently washed three times using TBST solution. Following primary incubation, membranes were incubated with the appropriate secondary antibodies. Protein bands were detected with Immobilon Western Chemilum HRP Substrate (cat. no. WBKLS0500; MilliporeSigma, USA) and images were acquired using an Amersham Imager 600 (Cytiva, USA). The experiment was performed for at least three technical replicates.

### Statistical analysis

Statistical analysis was performed using GraphPad Prism 8 (GraphPad Software, Inc.) and ImageJ software (National Institutes of Health). All values were presented as the mean ± SD. Student’s t-test (two-tailed) and ANOVA were used to compare groups for independent samples. *P* < 0.05 were considered to indicate a statistically significant. *p < 0.05, **p < 0.01, ***p < 0.001, ^ns^*p* > 0.05.

## Results

### CirSLC7A6 may affect the chemoresistance of ovarian cancer through regulation of miR-2682-5p and downstream gene SLC7A6

**Fig. 1:**
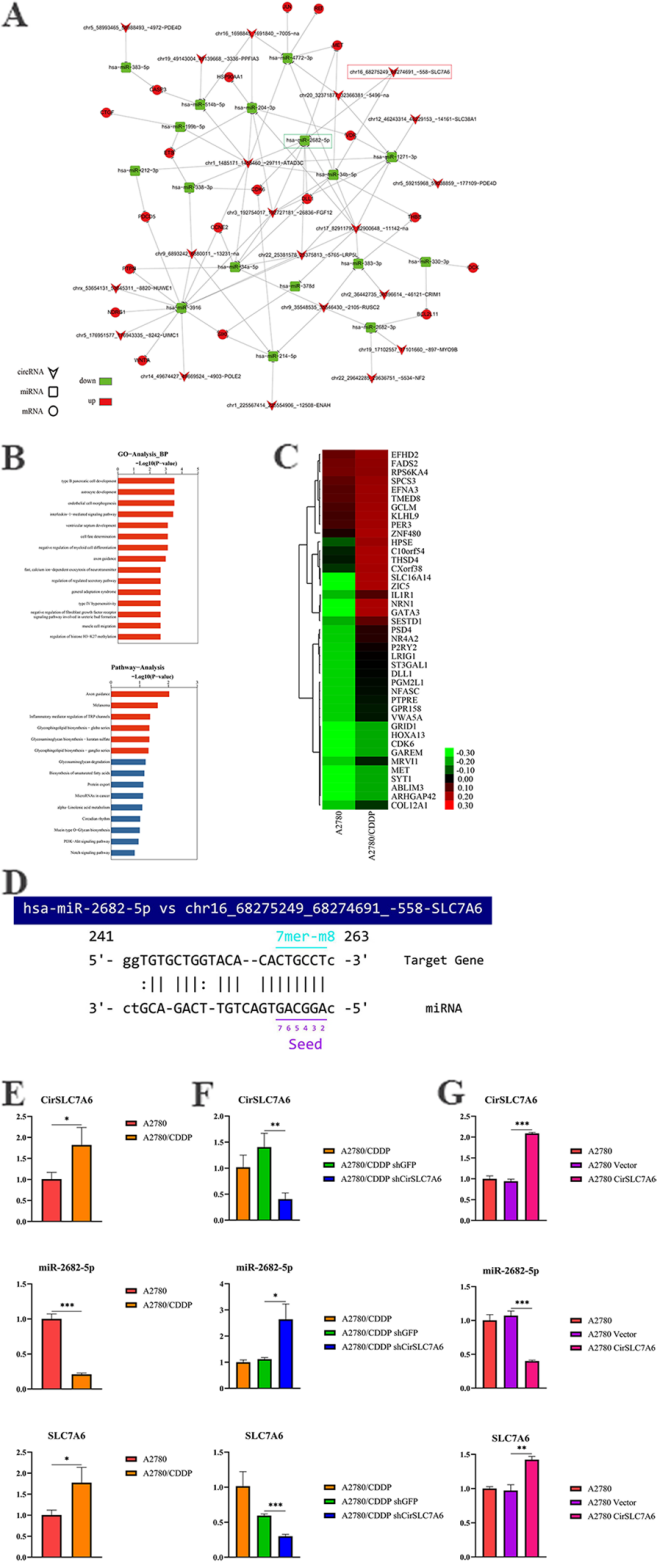
CirSLC7A6 may affect the chemoresistance of ovarian cancer through regulating miR-2682-5p and downstream gene SLC7A6. **A** Network of circRNAs and genes in A2780 and A2780/CDDP cells. **B** GO and KEGG pathway analysis.** C** Heatmap.** D** Binding sites of CirSLC7A6 and miR-2682-5p. Reverse transcription-quantitative PCR was carried out to detect the expression of CirSLC7A6, miR-2682-5p and SLC7A6 in (**E**) A2780 and A2780/CDDP cells, **(F)** A2780/CDDP shGFP and A2780/CDDP shCirSLC7A6 cells. And (**G**) A2780 Vector and A2780 CirSLC7A6 cells. circRNA, circular RNA; miRNA, microRNA; SLC7, solute carrier family 7; GO, gene ontology; KEGG, Kyoto Encyclopedia of Genes and Genomes

To investigate differentially expressed circRNAs in chemotherapy-sensitive and -resistant cells, high-throughput transcriptomic sequencing and bioinformatics analysis were performed. Results of the present study demonstrated that the expression of CirSLC7A6 was significantly increased in A2780/CDDP cells (Fig. 1A). Furthermore, CirSLC7A6 may be involved in the regulation of miR-2682-5p, and enriched biological functions mainly included cell proliferation, metabolism and adhesion (Fig. 1B-D). As shown in Fig. 1E, results of the RT-qPCR analysis demonstrated that CirSLC7A6 was markedly upregulated in A2780/CDDP cells, compared with A2780 cells (*P* < 0.05). MiR-2682-5p was downregulated in A2780/CDDP cells compared with that in A2780 cells (*P* < 0.05). Results of the present study demonstrated that the expression of CirSLC7A6 and miR-2682-5p were consistent with the results obtained from high-throughput transcriptomic sequencing. Moreover, results of the present study demonstrated that SLC7A6 was highly expressed in A2780/CDDP cells compared to A2780 cells (*P* < 0.05), which was consistent with the expression of CirSLC7A6. However, the opposite was found for the expression of miR-2682-5p. Therefore, CirSLC7A6 was identified as a molecular target for subsequent research on the chemoresistance of ovarian cancer. We hypothesized that CirSLC7A6 possesses a regulatory association with miR-2682-5p, and SLC7A6 may act as a downstream regulatory gene.

To verify the regulatory association between CirSLC7A6 and miR-2682-5p, and to further explore the mechanism of CirSLC7A6 regulating the chemoresistance of ovarian cancer, CirSLC7A6 was silenced in A2780/CDDP cells and overexpressed in A2780 cells. Transfection efficiency was verified using RT-qPCR. As shown in Fig. 1F, the expression of miR-2682-5p was significantly increased, while the expression of SLC7A6 was significantly decreased following knockdown of CirSLC7A6 (*P* < 0.05). However, overexpressing CirSLC7A6 exhibited the opposite effect on the expression of miR-2682-5p and SLC7A6 (*P* < 0.05; Fig. 1G). These results suggested that there is a regulatory association between CirSLC7A6 and miR-2682-5p, and SLC7A6 is a downstream regulatory gene. CirSLC7A6 may affect the uptake of amino acids, resulting in the chemoresistance of ovarian cancer cells via suppression of miR-2682-5p and activation of SLC7A6.

### CirSLC7A6 knockdown increases the anti-proliferative and pro-apoptotic effects of combined cisplatin and baicalein in resistant ovarian cancer cells

**Fig. 2:**
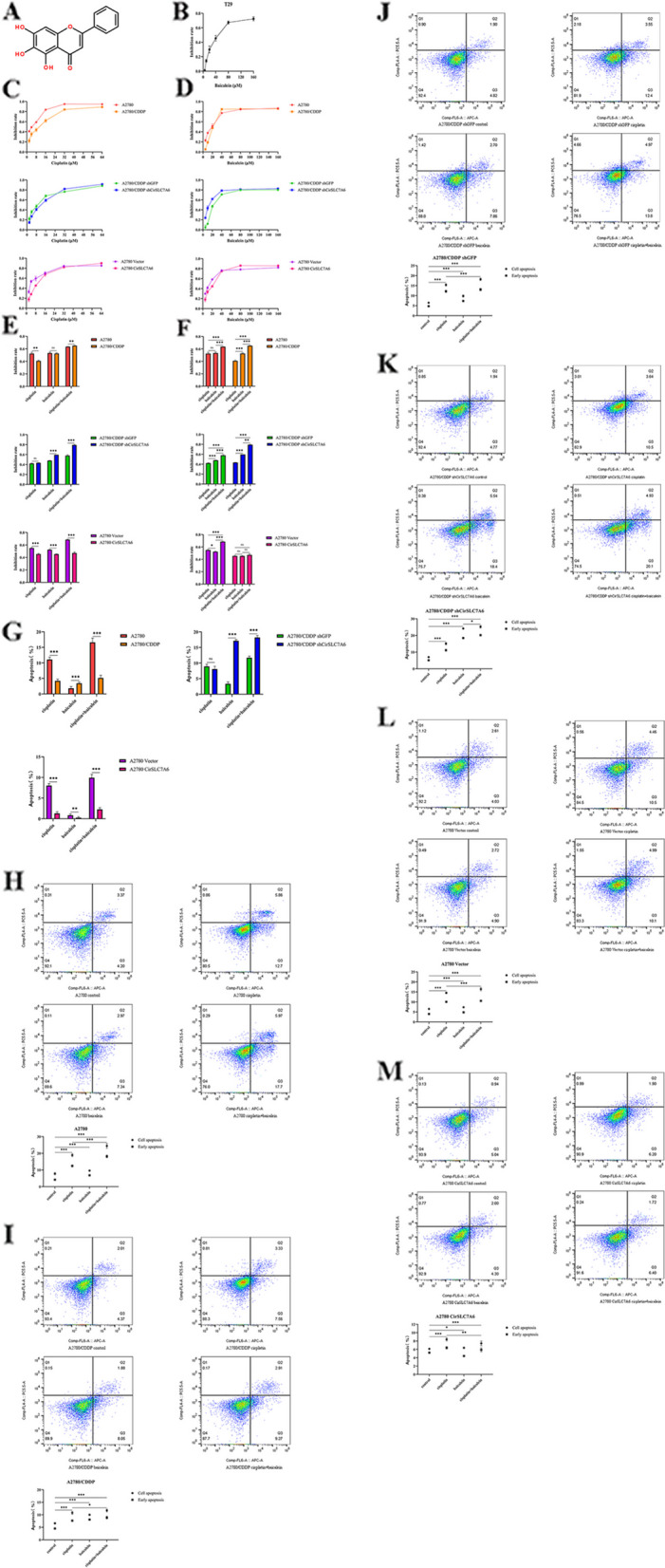
CirSLC7A6 knockdown increases the anti-proliferative and pro-apoptotic effects of the combined treatment of cisplatin and baicalein on resistant ovarian cancer cells.** A** Chemical structure of baicalein. **B** Baicalein inhibits the proliferation of T29 cells. **C** Cisplatin inhibits the proliferation of six ovarian cancer cell types. **D** Baicalein inhibits the proliferation of six ovarian cancer cell types. **E** Comparison of the inhibition of six ovarian cancer cell types treated with the same drug.** F** Comparison of the inhibition of six ovarian cancer cell types treated with different drugs. **G** Comparison of the apoptosis rates of six ovarian cancer cell types treated with the same drug. **H** Comparison of the apoptosis rate of A2780 cells treated with different drugs. **I** Comparison of the apoptosis rate of A2780/CDDP cells treated with different drugs. **J** Comparison of the apoptosis rate of A2780/CDDP shGFP cells treated with different drugs. **K** Comparison of the apoptosis rate of A2780/CDDP shCirSLC7A6 cells treated with different drugs.** L** Comparison of the apoptosis rate of A2780 Vector cells treated with different drugs.** M** Comparison of the apoptosis rate of A2780 CirSLC7A6 cells treated with different drugs. circRNA, circular RNA; SLC7, solute carrier family 7; shRNA, short hairpin RNA

To investigate whether baicalein suppresses the proliferation and promotes the apoptosis of ovarian cancer cells via the regulation of CirSLC7A6/miR-2682-5p/SLC7A6, a CCK-8 assay and flow cytometry were performed. The sensitivity of normal ovarian epithelial cells (T29) to baicalein was lower than that of ovarian cancer cells (Fig. 2B-D), suggesting that baicalein exerted a lower cytotoxicity in normal ovarian epithelial cells.

Results of the CCK-8 assay and flow cytometry (Fig. 2E-M) demonstrated that the sensitivity of A2780 cells to cisplatin was higher than that of A2780/CDDP cells, and cisplatin effectively attenuated the proliferation and promotes the apoptosis of A2780 cells. However, CirSLC7A6 knockdown did not increase the anti-proliferation and pro-apoptotic effects of cisplatin in A2780/CDDP cells, which indicated that knockdown of CirSLC7A6 may not improve cisplatin sensitivity. In addition, overexpression of CirSLC7A6 reduced the anti-proliferative and pro-apoptotic effects of cisplatin in A2780 cells and reduced cisplatin sensitivity. Baicalein exhibited similar anti-proliferative effects on A2780 and A2780/CDDP cells; however, the pro-apoptotic effects were higher in A2780/CDDP cells. CirSLC7A6 knockdown enhanced the anti-proliferative and pro-apoptotic effects of baicalein in A2780/CDDP cells. Overexpression of CirSLC7A6 attenuated the anti-proliferative and pro-apoptotic effects of baicalein in A2780 cells. Compared with A2780 cells, a combination of cisplatin and baicalein exhibited stronger anti-proliferative effects and weaker pro-apoptotic effects in A2780/CDDP cells. Knockdown of CirSLC7A6 significantly promoted the anti-proliferative and pro-apoptotic effects of the combination of cisplatin and baicalein in A2780/CDDP cells. Overexpression of CirSLC7A6 exerted the opposite effect in A2780 cells. A combination of cisplatin and baicalein was superior to either cisplatin or baicalein treatment alone in suppressing cell proliferation and inducing cell apoptosis in A2780 and A2780/CDDP cells. These results suggested that baicalein may inhibit cell proliferation in coordination with cisplatin. CirSLC7A6 knockdown markedly increased the chemosensitivity of baicalein, and significantly improved the anti-proliferative and pro-apoptotic effects of the combination of cisplatin and baicalein in A2780/CDDP cells. However, overexpression of CirSLC7A6 significantly reduced the chemosensitivity of baicalein.

### Baicalein suppresses the metastasis of resistant ovarian cancer via the regulation of CirSLC7A6

**Fig. 3:**
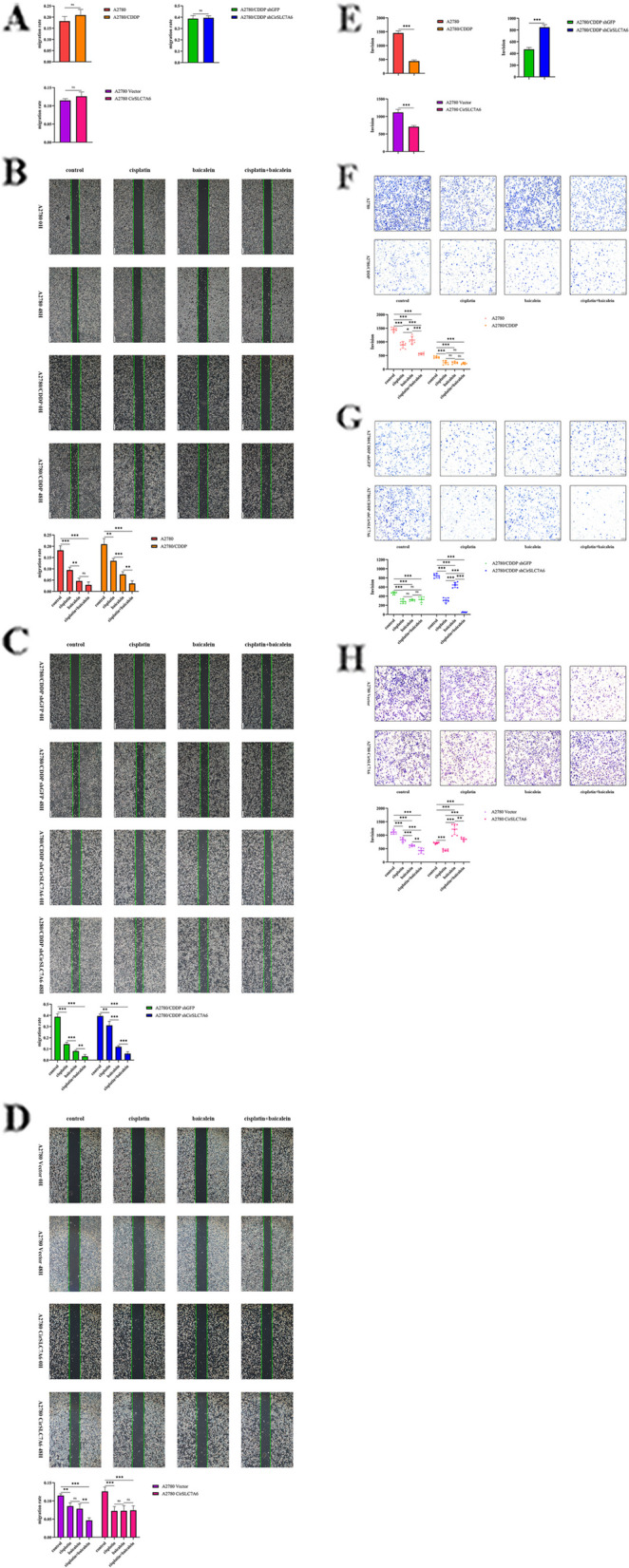
Baicalein suppresses the migration and invasion of resistant ovarian cancer cells through regulating CirSLC7A6.** A** Intercellular comparison of the migration rate.** B** Intracellular comparison in A2780 and A2780/CDDP cells.** C** Intracellular comparison in A2780/CDDP shGFP and A2780/CDDP shCirSLC7A6 cells.** D** Intracellular comparison in A2780 Vector and A2780 CirSLC7A6 cells. **E** Intercellular comparison of cell invasion.** F** Intracellular comparison in A2780 and A2780/CDDP cells.** G** Intracellular comparison in A2780/CDDP shGFP and A2780/CDDP shCirSLC7A6 cells.** H** Intracellular comparison in A2780 Vector and A2780 CirSLC7A6 cells. circRNA, circular RNA; SLC7, solute carrier family 7; shRNA, short hairpin RNA

To investigate whether baicalein inhibits cell metastasis in ovarian cancer, wound healing and Transwell assays were conducted. As shown in Fig. 3A, there was no significant difference in cell mobility between A2780 and A2780/CDDP cells (*P* > 0.05). Similarly, there was no significant difference in cell mobility between A2780/CDDP shCirSLC7A6 cells and A2780/CDDP shGFP cells (*P* > 0.05). There was no significant difference in cell mobility between A2780 CirSLC7A6 cells and A2780 Vector cells (*P* > 0.05). These results suggested that CirSLC7A6 expression exhibited no significant effects on the migration of ovarian cancer cells.

As shown in Fig. 3B, in A2780 cells, the cell migration of the control group was higher than that of all treatment groups (*P* < 0.05). The cell mobility of the cisplatin group was higher than that of the baicalein group (*P* < 0.05). There was no statistical difference between the baicalein group and the group treated with a combination of cisplatin and baicalein (*P* > 0.05). These results indicated that the inhibitory effects of baicalein were stronger than cisplatin; however, a combination of the two drugs did not inhibit the migration of A2780 cells. In A2780/CDDP cells, cell migration of the control group was higher than that of all treatment groups (*P* < 0.05). Cell mobility of the cisplatin group was higher than that of the baicalein group (*P* < 0.05). In addition, cell migration in the baicalein group was higher than the group treated with a combination of both drugs (*P* < 0.05). These results revealed that baicalein may inhibit the migration of A2780/CDDP cells more effectively than cisplatin. Cisplatin and baicalein potentially synergically inhibit the migration of A2780/CDDP cells.

As shown in Fig. 3C, in A2780/CDDP shCirSLC7A6 cells, migration of the control group was higher than that of all treatment groups (*P* < 0.05). The cell mobility of the cisplatin group was significantly higher than that of the baicalein group (*P* < 0.05). Cell migration of the baicalein group was higher than that of the group treated with a combination of both drugs (*P* < 0.05). These results indicated that migration inhibition is significantly enhanced following treatment with a combination of both drugs, compared with groups treated with either baicalein or cisplatin alone in A2780/CDDP shCirSLC7A6 cells.

As shown in Fig. 3D, in A2780 CirSLC7A6 cells, migration in the control group was 12.6%, which was higher than that of all treatment groups (*P* < 0.05). However, there was no statistical difference among cisplatin, baicalein and the combined treatment groups (*P* > 0.05). These results indicated that a combination of both drugs may not inhibit the migration of A2780 CirSLC7A6 cells.

As shown in Fig. 3E, A2780 cells were more invasive than A2780/CDDP cells (*P* < 0.05), and the invasion of A2780/CDDP shCirSLC7A6 cells was significantly increased, compared with A2780/CDDP shGFP cells (*P* < 0.05). However, the invasion of A2780 CirSLC7A6 cells was significantly decreased compared with A2780 Vector cells (*P* < 0.05). The results demonstrated that invasion of A2780/CDDP cells is lower than that of A2780 cells. CirSLC7A6 knockdown enhanced the invasion of A2780/CDDP cells, while overexpression of CirSLC7A6 attenuated the invasion of A2780 CirSLC7A6 cells.

As shown in Fig. 3F, in A2780 cells, invasion of the control group was higher than that of all treatment groups (*P* < 0.05). In addition, invasion of the cisplatin group was lower than that of the baicalein group (*P* < 0.05), while invasion of groups treated with either cisplatin or baicalein alone was higher than that of the group treated with a combination of both drugs (*P* < 0.05). Treatment of both cisplatin and baicalein in combination inhibited the invasion at a higher level, than either drug alone. In A2780/CDDP cells, the invasion of the control group was higher than that of all treatment groups (*P* < 0.05), but there was no statistical difference among treatment groups (*P* > 0.05). Cisplatin and baicalein exerted similar inhibitory effects on the invasion of A2780/CDDP cells, and a combination of both drugs did not synergically inhibit cell invasion.

As shown in Fig. 3G, in A2780/CDDP shCirSLC7A6 cells, invasion of the control group was higher than that of all treatment groups (*P* < 0.05), and invasion of the cisplatin group was lower than that of the baicalein group (*P* < 0.05). In addition, invasion in groups treated with either cisplatin or baicalein alone was significantly higher than that of the group treated with a combination of both drugs (*P* < 0.05). The results demonstrated that CirSLC7A6 knockdown enhanced the synergistic inhibitory effects of cisplatin and baicalein on the invasion of A2780/CDDP cells.

As shown in Fig. 3H, in A2780 CirSLC7A6 cells, invasion of the control group was higher than that of the cisplatin group (*P* < 0.05), and invasion of the control group was lower than that of the baicalein or combined treatment groups (*P* < 0.05). Invasion of the baicalein group was significantly higher than that of the combined treatment group (*P* < 0.05). These results suggested that overexpression of CirSLC7A6 attenuates the inhibitory effects of baicalein and a combination of both drugs on the invasion of A2780 cells.

### CirSLC7A6 knockdown enhances the synergistic inhibitory effects of cisplatin and baicalein on tumor growth in ovarian cancer

**Fig. 4:**
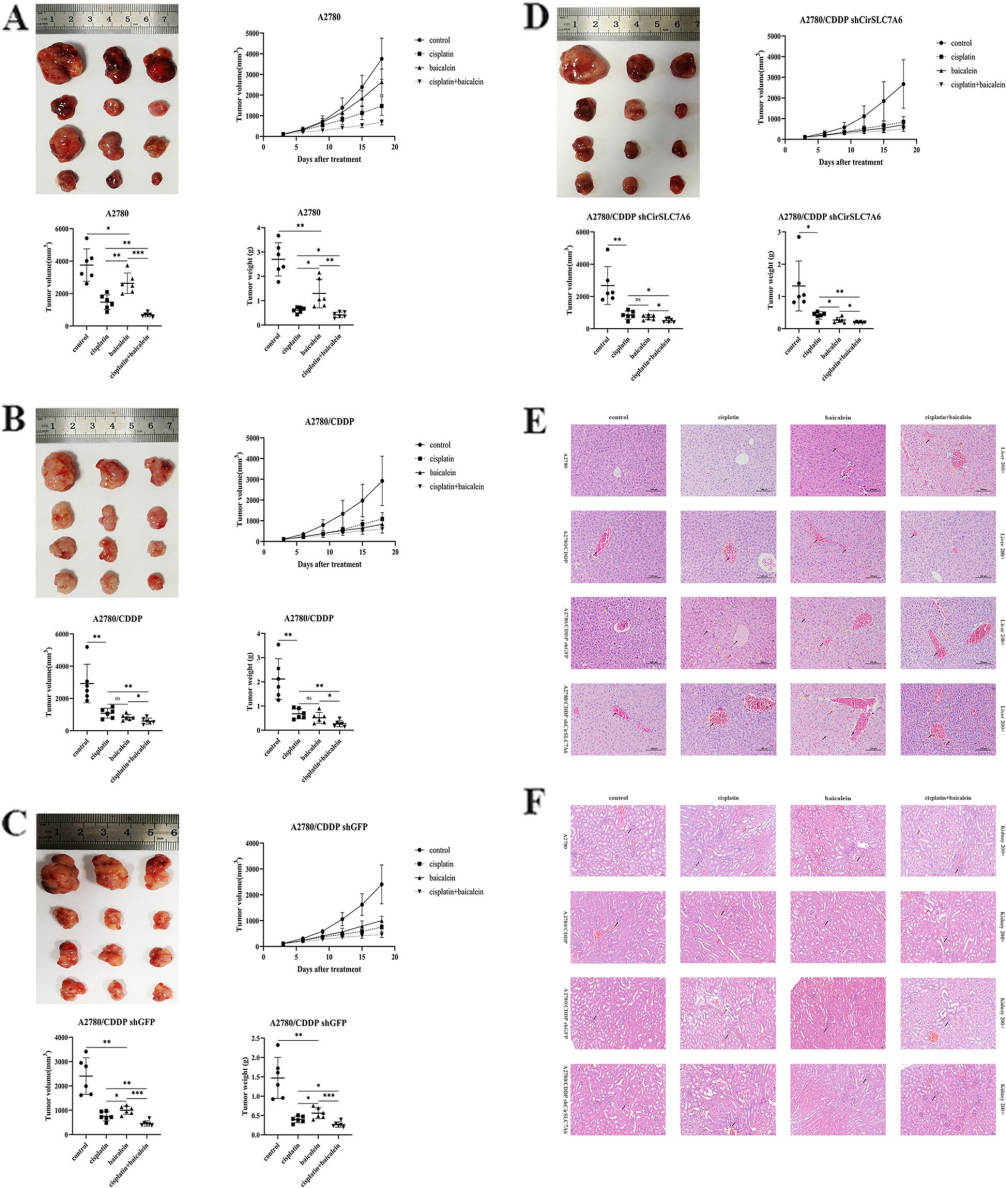
CirSLC7A6 knockdown enhances the synergistic inhibitory effects of cisplatin and baicalein on the tumor growth of ovarian cancer.** A** Tumors subcutaneously transplanted with A2780 cells.** B** Tumors subcutaneously transplanted with A2780/CDDP cells. **C** Tumors subcutaneously transplanted with A2780/CDDP shGFP cells. **D** Tumors subcutaneously transplanted with A2780/CDDP shCirSLC7A6 cells. **E–F** Cisplatin plus baicalein causes no injury in the liver or kidney of mice transplanted with A2780/CDDP shCirSLC7A6 cells. circRNA, circular RNA; SLC7, solute carrier family 7; shRNA, short hairpin RNA

To further verify the chemosensitivity of baicalein through the regulation of CirSLC7A6/miR-2682-5p/SLC7A6 in ovarian cancer cells, tumor xenografts were established. Consistent with the previous results of the present study, as shown in Fig. 4A, in the tumor model subcutaneously transplanted with A2780 cells, the tumor weight of the control group was higher than that of the treatment groups (*P* < 0.05). The tumor weight of the cisplatin group was significantly lower than that of the baicalein group (*P* < 0.05). The tumor weight of the combined treatment group was lower than that of either the cisplatin or baicalein groups (*P* < 0.05). In addition, the tumor volume of the control group was larger than that of the treatment groups (*P* < 0.05). The tumor volume of the cisplatin group was smaller than that of the baicalein group (*P* < 0.05). The tumor volume of the combined treatment group was significantly lower than that of either the cisplatin or baicalein groups (*P* < 0.05). These results suggested that cisplatin exhibited an improved inhibition on tumor growth than baicalein. A combination of cisplatin and baicalein synergically suppressed tumor growth.

As shown in Fig. 4B, in the tumor model subcutaneously transplanted with A2780/CDDP cells, the tumor weight of the control group was higher than that of the treatment groups (*P* < 0.05). There was no statistical difference in tumor weight between the cisplatin and baicalein groups (*P* > 0.05). The tumor weight of the combined treatment group was significantly lower than that of either the cisplatin or baicalein groups (*P* < 0.05). In addition, the tumor volume of the control group was larger than that of the treatment groups (*P* < 0.05). There was no significant difference in tumor volume between the cisplatin and baicalein groups (*P* > 0.05). The tumor volume of the combined treatment group was significantly smaller than that of either the cisplatin or baicalein groups (P < 0.05). These data indicated that cisplatin and baicalein exerted similar inhibitory effects on tumor growth. The combination of cisplatin and baicalein synergically inhibited tumor growth.

As shown in Fig. 4D, in the tumor model subcutaneously transplanted with A2780/CDDP shCirSLC7A6 cells, the tumor weight of the control group was higher than that of the treatment groups (*P* < 0.05). The tumor weight of the cisplatin group was higher than that of the baicalein group (*P* < 0.05). The tumor weight of the combined treatment group was significantly lower than that of the cisplatin and baicalein groups (*P* < 0.05). In addition, the tumor volume of the control group was larger than that of the treatment groups (*P* < 0.05), but there was no significant difference in tumor volume between the cisplatin and baicalein groups (*P* > 0.05). The tumor volume of the combined treatment group was significantly lower than that of the cisplatin and baicalein groups (*P* < 0.05). Baicalein exhibited a higher inhibition on tumor growth than cisplatin. The combination of cisplatin and baicalein exerted significantly synergistic effects.

To identify the toxicity of cisplatin and baicalein on the liver and kidney, H&E staining was performed. H&E staining of the liver (Fig. 4E) demonstrated that in the tumor model subcutaneously transplanted with A2780 cells, there was no congestion in the control or cisplatin groups. Notably, congestion occurred in the baicalein and combined treatment groups, and it was more severe in the combined treatment group, suggesting that the combined treatment of cisplatin and baicalein may increase toxicity to the liver. In the tumor model subcutaneously transplanted with A2780/CDDP cells, congestion in treatment groups was more severe than in the control group. Congestion in the baicalein group was more severe than that of the combined treatment group, which indicated that the toxicity of the combined treatment of cisplatin and baicalein in the liver may be weakened. In the tumor model subcutaneously transplanted with A2780/CDDP shCirSLC7A6 cells, congestion in the treatment groups was more severe than in the control group. There was no significant difference among treatment groups, indicating that the combined treatment did not increase liver injury.

H&E staining of the kidney (Fig. 4F) demonstrated that in the tumor model subcutaneously transplanted with A2780 cells, there was no renal tubular expansion in the control or baicalein groups. Notably, renal tubular expansion occurred in the cisplatin and combined treatment groups. These findings demonstrated that baicalein was less toxic on the kidney than cisplatin or a combination of cisplatin and baicalein. However, the combined treatment of cisplatin and baicalein did not increase renal toxicity. In the tumor model subcutaneously transplanted with A2780/CDDP cells, there was no renal tubular expansion in the control group. Notably, renal tubular expansion in the cisplatin and combined treatment groups was more severe than that in the baicalein group. These findings demonstrated that cisplatin and a combination of cisplatin and baicalein exerted increased toxicity in the kidney. However, the combined treatment of both drugs did not increase renal toxicity. In the tumor model subcutaneously transplanted with A2780/CDDP shCirSLC7A6 cells, renal tubular expansion was only found in the cisplatin group, indicating that the toxicity of cisplatin in the kidney was greater than that of either baicalein or a combined treatment of cisplatin and baicalein. Notably, baicalein alleviated the renal toxicity of cisplatin.

### Baicalein inhibits the proliferation and metastasis of resistant ovarian cancer cells through regulation of CirSLC7A6

**Fig. 5:**
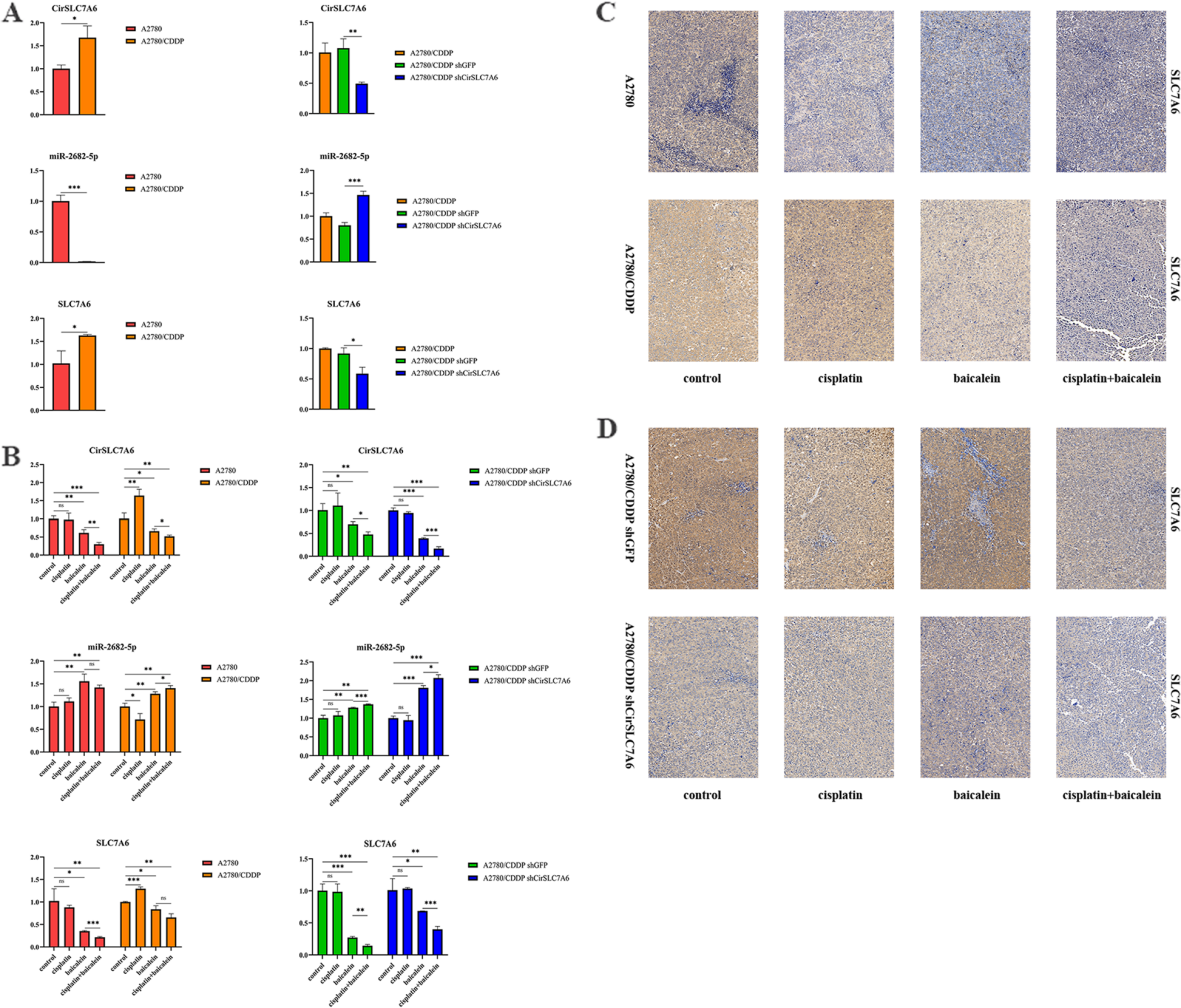
Reverse transcription-quantitative PCR indicated that the expression levels of CirSLC7A6 were significantly decreased, and a combination of cisplatin and baicalein downregulated the expression of CirSLC7A6 in the A2780/CDDP shCirSLC7A6 cell xenograft.** A** Intercellular comparison.** B** Intracellular comparison. **C-D** Immunohistochemical results demonstrated that the expression levels of SLC7A6 were significantly decreased and the combined treatment of cisplatin and baicalein downregulated the expression of SLC7A6 in the A2780/CDDP shCirSLC7A6 cell xenograft. circRNA, circular RNA; SLC7, solute carrier family 7; shRNA, short hairpin RNA

RT-qPCR and immunohistochemistry were used to detect the expression of CirSLC7A6, miR-2682-5p and SLC7A6 in tumor tissues. As shown in Fig. 5A, results of the RT-qPCR analysis indicated that the expression of CirSLC7A6 and SLC7A6 in the A2780/CDDP cell xenograft were notably higher than those in the A2780 cell xenograft; however, miR-2682-5p expression was decreased (*P* < 0.05). The expression levels of CirSLC7A6 and SLC7A6 in the A2780/CDDP shCirSLC7A6 cell xenograft were significantly lower than those in the A2780/CDDP shGFP cell xenograft; however, miR-2682-5p was increased (*P* < 0.05). As shown in Fig. 5B, in the A2780, A2780/CDDP and A2780/CDDP shCirSLC7A6 cell xenografts, baicalein and the combined treatment of both drugs reduced the expression of CirSLC7A6 and SLC7A6, and increased the expression of miR-2682-5p (*P* < 0.05). Notably, cisplatin promoted the expression of CirSLC7A6 and SLC7A6, and inhibited the expression of miR-2682-5p in the A2780/CDDP cell xenograft (*P* < 0.05).

As shown in Fig. 5(C-D), results of the immunohistochemical analysis demonstrated that the expression of SLC7A6 was significantly higher in the A2780/CDDP cell xenograft than that in the A2780 cell xenograft (*P* < 0.05). The expression of SLC7A6 was markedly lower in the A2780/CDDP shCirSLC7A6 cell xenograft than in the A2780/CDDP shGFP cell xenograft (*P* < 0.05). In the A2780, A2780/CDDP and A2780/CDDP shCirSLC7A6 cell xenograft, the combined treatment of both drugs suppressed the expression of SLC7A6 (*P* < 0.05). Animal experiments further confirmed that CirSLC7A6 may regulate the expression of miR-2682-5p and SLC7A6, and CirSLC7A6 knockdown enhanced the inhibitory effects of the combined treatment of both drugs on SLC7A6.

### Baicalein attenuates PI3K/Akt/mTOR and Erk/p38 MAPK pathways and sensitizes cisplatin via the regulation of CirSLC7A6 in resistant ovarian cancer cells

**Fig. 6:**
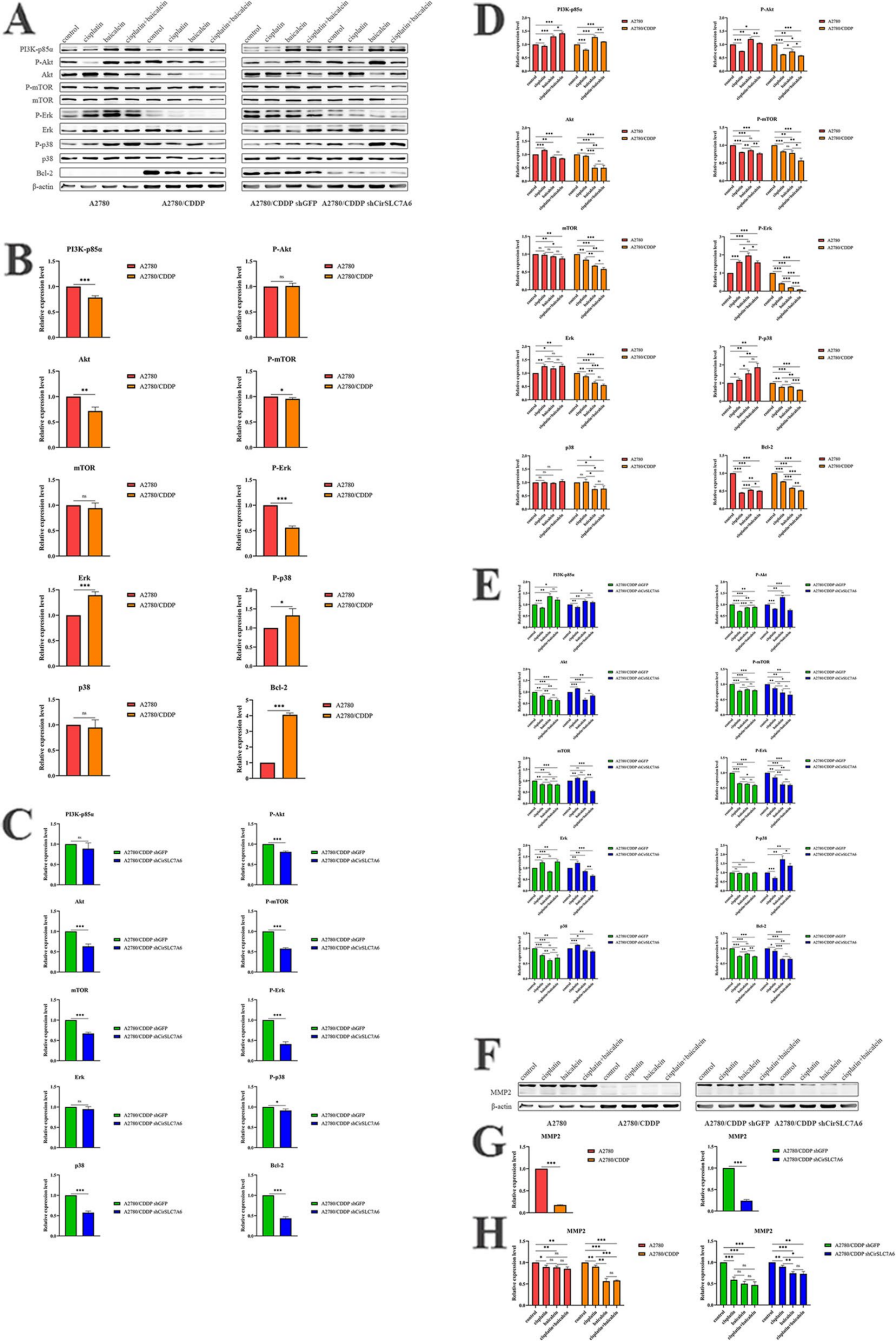
Baicalein inhibits the proliferation of resistant ovarian cancer cells through regulating CirSLC7A6. **A** Protein bands.** B** The quantitative histograms of the intercellular comparison between A2780 and A2780/CDDP cells.** C** The quantitative histograms of the intercellular comparison between A2780/CDDP shGFP and A2780/CDDP shCirSLC7A6 cells.** D** The quantitative histograms of the intracellular comparison in A2780 and A2780/CDDP cells.** E** The quantitative histograms of the intracellular comparison in A2780/CDDP shGFP and A2780/CDDP shCirSLC7A6 cells. Baicalein inhibits the metastasis of resistant ovarian cancer cells through regulating CirSLC7A6.** F** Protein bands. **G** The quantitative histograms of the intercellular comparison. **H** The quantitative histograms of the intracellular comparison. circRNA, circular RNA; SLC7, solute carrier family 7; shRNA, short hairpin RNA

To further elucidate the role of baicalein in the cisplatin sensitivity of resistant ovarian cancer cells, western blot analysis was carried out to measure the protein expression levels of PI3K-p85α, P-Akt, Akt, P-mTOR, mTOR, P-Erk, Erk, P-p38, p38 and Bcl-2 (Fig. 6A-C). These results demonstrated that knockdown of CirSLC7A6 potentially inhibits the phosphorylation of Akt, mTOR, Erk and p38, and reduces the activity of Bcl-2, thereby inhibiting cell proliferation and promoting cell apoptosis.

As shown in Fig. 6D, in A2780 cells, all treatment options inhibit the phosphorylation of mTOR and decrease the expression of Bcl-2, thereby suppressing proliferation and promoting apoptosis of A2780 cells. In A2780/CDDP cells, all treatment options inhibited the phosphorylation of Akt, mTOR, Erk and p38, and decreased the activity of Bcl-2, thereby inhibiting proliferation and promoting apoptosis of A2780/CDDP cells.

As shown in Fig. 6E, in A2780/CDDP shCirSLC7A6 cells, cisplatin not only inhibits the activation of PI3K/Akt/mTOR signaling, but also inhibits the expression of P-Erk, P-p38 and Bcl-2. On the other hand, baicalein reduced the phosphorylation of mTOR and Erk, and reduced the overall expression of Bcl-2. By contrast, the combined treatment of cisplatin and baicalein exerted anti-tumor effects through inhibition of the phosphorylation of Akt, mTOR and Erk, and the expression of Bcl-2.

These findings suggested that CirSLC7A6 knockdown inhibits the phosphorylation of Akt, mTOR, Erk and p38, and reduces the expression of Bcl-2; however, it does not increase the sensitivity of ovarian cancer cells to cisplatin. The mechanism of resistance to cisplatin is complex. Although CirSLC7A6/miR-2682-5p/SLC7A6 and downstream pathways are suppressed, compensation mechanisms may be activated. CirSLC7A6 knockdown alone did not improve the sensitivity of cisplatin. A combination of both cisplatin and baicalein may inhibit the CirSLC7A6/miR-2682-5p/SLC7A6 pathway and block the compensation of amino acid transportation. This may increase the intracellular cisplatin concentration; thus, inhibiting the phosphorylation of Akt, mTOR and Erk, and decreasing the level of Bcl-2, leading to ovarian cancer cells apoptosis.

To investigate the molecular mechanism underlying baicalein-regulated cell metastasis, western blot analysis was carried out to determine the protein expression levels of MMP2 (Fig. 6F-H). These results indicated that CirSLC7A6 knockdown inhibits the expression of MMP2. The combination of both cisplatin and baicalein further significantly reduced the protein expression level of MMP2, and subsequently suppressed the metastasis of ovarian cancer cells.

## Discussion

EOC is the most lethal gynecological malignant tumor. At present, cytoreductive surgery combined with platinum-based chemotherapy is the standard treatment for EOC. However, the recurrence rate is as high as 85%, and chemoresistance gradually develops, leading to a poor prognosis [26]. Baicalein may activate or inactivate specific pathways in cells through regulating non-coding RNAs; thus, inducing apoptosis or increasing drug sensitivity of resistant cells [27–29]. Baicalein inhibits cervical cancer cell growth through regulating circHIAT1/miR-19a-3p, which targets the PI3K/Akt/mTOR pathway [27]. Moreover, baicalein suppresses hepatocellular carcinoma progression through targeting the lncRNA/miR-4443/Akt1 network [28]. Baicalein also suppresses prostate cancer proliferation through inhibiting lncRNA, decreasing PCGEM1 expression and inducing cell autophagy [29]. CircRNA regulates biological processes, such as proliferation, apoptosis, metabolism, migration and chemoresistance of tumor cells, through interacting with miRNA and mRNA [30]. CircATRNL1 absorbs miR-378, activates Smad4, and further decreases the activities of Akt, Cyclin D1, MMP2 and MMP9, thereby inhibiting proliferation, angiogenesis and metastasis of ovarian cancer cells [31]. CircRNA CDR1as enhances the cisplatin sensitivity of ovarian cancer cells through regulating the miR-1270/SCAI axis [32].

CircATL2 promotes the paclitaxel resistance of ovarian cancer cells through regulating miR-506-3p and upregulating nuclear factor IB expression. CircATL2 knockdown promotes apoptosis in paclitaxel-resistant ovarian cancer cells, thereby reversing paclitaxel resistance [33]. SLC7A6 mediates the uptake of arginine, leucine and glutamine, and transports amino acids to exchange arginine and glutamine both intra- and extracellularly [34–36]. SLC7A6 is upregulated in hepatocellular carcinoma, leading to a significant increase in the uptake of amino acids, which further activates the mTOR signaling cascade and promotes tumor formation. Downregulation of SLC7A6 inhibits the proliferation and invasion of hepatocellular carcinoma [37, 38]. SLC7A6 is upregulated in resistant prostate cancer. Knockdown of SLC7A6 leads to significant leucine uptake inhibition and cell growth inhibition through the mTOR pathway [39].

Results of the present study demonstrated that CirSLC7A6 suppressed miR-2682-5p and further activated SLC7A6 in A2780/CDDP cells. Knockdown of CirSLC7A6 alone did not improve the cisplatin sensitivity of ovarian cancer cells, despite inactivation of the PI3K/Akt/mTOR and Erk/p38 MAPK pathways. Other transporters not investigated in the present study may compensate for the loss of SLC7A6, contributing to the maintenance of cisplatin resistance of ovarian cancer. Moreover, overexpression of CirSLC7A6 activated SLC7A6 and its downstream signaling pathways in A2780 cells, thereby improving chemoresistance. However, silencing CirSLC7A6 effectively increased the chemosensitivity of baicalein, and significantly improved the anti-proliferative and pro-apoptotic effects of the combination of cisplatin and baicalein in A2780/CDDP cells. Overexpression of CirSLC7A6 exerted the opposite effect in A2780 cells. Cisplatin and baicalein may inhibit the CirSLC7A6/miR-2682-5p/SLC7A6 axis, and block the compensation of amino acid transportation simultaneously, further inactivating the PI3K/Akt/mTOR and Erk/p38 MAPK pathways.

Cadherin mediates calcium-dependent cell–cell adhesion. E-cadherin is mainly distributed in epithelial tissues. The downregulation of E-cadherin reduces the strength of cell adhesion within tissues, leading to increased cell motility [40]. Cancer cells degrade the extracellular matrix barrier through secreting MMP2 and MMP9, and subsequently bind to adhesion receptors on the basement membrane, promoting cell mobility [40]. Results of previous studies have demonstrated that the PI3K/Akt/mTOR and MAPK pathways in ovarian cancer affect cell metastasis through regulating the expression of E-cadherin, CAM, MMP and tumor angiogenic factors [41–44]. Results of the present study demonstrated that CirSLC7A6 knockdown significantly enhanced the synergistic inhibitory effects of cisplatin and baicalein on the migration and invasion of A2780/CDDP cells. In addition, overexpression of CirSLC7A6 improved the invasion of A2780 cells mediated by cisplatin and baicalein. Notably, cisplatin and baicalein may suppress MMP2 protein expression and cell metastasis of resistant ovarian cancer through regulating the CirSLC7A6/miR-2682-5p/SLC7A6 axis.

Cisplatin is mainly cleared by the kidney. Nephrotoxicity induced by cisplatin is dose-limited and kidney injury is the most common manifestation. Exposure of kidney tubules to cisplatin activates complex signaling pathways, leading to cell damage and death [45]. Organic transporters and amino acid transporters increase the uptake of drugs by the kidney, reduce the excretion of drugs, and significantly increase the concentration of cisplatin. This leads to DNA damage, mitochondrial dysfunction, activation of apoptotic pathways and production of reactive oxygen species; thus, resulting in nephrotoxicity. Cisplatin-induced hepatotoxicity exhibits a similar mechanism [46]. Baicalein possesses anti-inflammatory effects and may alleviate the damage of liver and kidney function caused by chemotherapeutic agents, through reducing oxidative stress [47–49]. Results of the present study demonstrated that following CirSLC7A6 knockdown, the combined treatment of cisplatin and baicalein did not increase liver and kidney injury in ovarian cancer. Baicalein may reduce oxidative stress levels and alleviate damage of the liver and kidney caused by cisplatin, through regulating CirSLC7A6.

## Conclusions

Collectively, results of the present study demonstrated that baicalein improves the sensitivity of ovarian cancer cells to cisplatin via inhibiting CirSLC7A6/miR-2682-5p/SLC7A6, and further inactivating PI3K/Akt/mTOR and Erk/p38 MAPK pathways. Notably, additional damage of the liver and kidney is not observed when cisplatin is used in combination with baicalein. In addition, baicalein cooperating with cisplatin suppresses MMP2 protein expression and cell metastasis of resistant ovarian cancer cells, through regulation of CirSLC7A6/miR-2682-5p/SLC7A6.

## Data Availability

The datasets analyzed during the present study are available from the corresponding author on reasonable request.
